# Multi-level barriers to early detection of breast cancer among rural midlife women in Tanzania: A qualitative case study

**DOI:** 10.1371/journal.pone.0297798

**Published:** 2024-02-29

**Authors:** Tumaini Nyamhanga, Rosemary W. Eustace, Janeth Philip Makoye, Katunzi Mutalemwa

**Affiliations:** 1 Department of Developmemt Studies, Muhimbili University of Health and Allied Sciences (MUHAS), Dar es Salaam, Tanzania; 2 School of Nursing, Kinesiology, and Health Sciences, College of Health, Education and Human Services (CHEH), Wright State University, Dayton, OH, United States of America; 3 Health Department, Ileje District Council, Songwe Region, Tanzania; 4 Doctors with Africa CUAMM, Iringa, Tanzania; The University of Dodoma, UNITED REPUBLIC OF TANZANIA

## Abstract

**Background:**

Breast cancer is the second most common cause of cancer mortality among women in Tanzania and thus, early detection and treatment methods are central to improving breast cancer outcomes. However, in low- and middle-income countries in Sub-Saharan Africa, the survival rates remains low due to late presentation. Hence, a significant number of deaths could be prevented if barriers and facilitators to early detection are known.

**Purpose:**

This qualitative case descriptive study explored the possible barriers to awareness and early breast cancer diagnostic services among midlife women in rural Tanzania.

**Methods:**

Ten key informant interviews with health systems managers and community health workers and eight focus group discussions with women aged 40–65 years and their spouses were conducted to elicit the study data conducted from July to August 2021.

**Results:**

The data revealed nine themes describing the barriers to early detection methods across five Socio-Ecological levels of influence, namely: 1) limited knowledge and 2) witchcraft beliefs (*individual level*); 3) limited male support (*interpersonal level*); 4) age and gender factors and 5) procrastination (*community level*) 6) limited availability of services 7) emphasis of curative over preventive care (*institutional level*); 8) poverty/inability to pay and 9) limitations of health insurance (*societal/policy level*).

**Conclusions:**

The study findings suggest a need to further the design, implementation and evaluation of evidence-based community breast health awareness and education interventions to promote early detection of breast cancer in Tanzania. Specifically, the study highlights the need to address multiple level determinants of influence in breast cancer control as part of the country’s Community Health Strategy.

## Introduction

Breast cancer is a devastating global health problem, particularly in sub-Saharan Africa (SSA). Compared to high-income nations, Breast cancer mortality rates in Sub-Saharan Africa (SSA) are disproportionately high, despite the region’s disease frequency appearing to be lower [1]. Although the disease incidence in SSA seems lower, mortality rates are disproportionately high in comparison to high-income countries. In Tanzania, Breast cancer is the second most common cancer, accounting for 14.4% of all newly diagnosed cancer patients [2]. It also ranks second in terms of cancer-related deaths among women. By 2030, an 82% increase in new instances of breast cancer is anticipated [2].

Globally, early detection and treatment methods are central to improving breast cancer outcomes. However, in low- and middle-income countries (LMICs) like those in Sub-Saharan Africa, the survival rates remain low due to late presentation that is attributed to inadequate or lack of access to early detection methods and treatment [3–6] such as mammography [7–9]. Thus, low cost, early detection strategies based on public health education related to awareness of risks, education about breast self-examination (BSE), clinical breast examination (CBE), and a well-trained health care workforce are considered the first line of control in improving breast cancer outcomes and survival rates [10–12]. In Tanzania, there are well-established health care system structures for health care provision from the least resourced community-level structures to specialized national hospitals. As of November, 2023, there were 7744 Dispensaries; 1115 Health Centers; 171 District Hospitals; 28 Regional Referral Hospitals; 5 Zonal Referral Hospitals; and 1 National Hospital [13]. In addition, Tanzania has **National Guidelines** for Early Diagnosis of Breast Cancer and Referral for Treatment [14]. However, due to shortage of skilled human resources and inadequacy/ absence of essential medical supplies for diagnosis and treatment of breast cancer, not all levels of health care perform as prescribed in the guidelines. The recent national assessment conducted in 2017 [15] revealed lower health facilities (from dispensaries to the district hospitals) lack patient information and do not perform routine clinical breast examination; while regional referral hospitals have some capacity for diagnosis and surgical management. On the other hand, Zonal consultant hospitals (for example, Bugando Medical Centre) and Muhimbili National Hospital are better equipped with facilities for diagnosis and surgical management of breast cancer. These higher-level hospitals do provide access to chemotherapy when available. A full range of cancer treatment is offered at Ocean Road Cancer Institute (ORCI), which is a National referral hospital specifically for treatment of cancer.

Absence of consistent breast cancer awareness [16] and early detection methods in the region [2], suggest that women themselves should be proactive to seek care from the primary health facilities and then follow the referral system, if so recommended, to the better equipped Zonal and National Hospitals. The earlier one starts the journey the better. However, geographic distance to services is a significant barrier to timely care, especially for rural populations from which more than 80% of women with breast cancer are lately diagnosed [15]. To address this gap, intermittent efforts have been made to facilitate availability of early prevent methods through outreach services conducted by the Tanzania’s Ministry of Health, and Clinical Breast Examination (CBE) campaigns conducted by Non-Governmental Organizations (NGOs) such as Tanzania’s Medical Women Association (MEWATA). Unfortunately, most Tanzania women suffering from breast cancer continue to present with late diagnoses, suggesting existence of multiple barriers to early diagnosis and treatment [3–6]. Therefore, identifying the barriers and facilitators to early detection of breast cancer in LMICs [11] that cut across individual, interpersonal, organizational, community and health system barriers is needed for effective design and implementation of early detection methods [17]. However, few studies in the region have taken a multilevel approach to investigate barriers to early detection of breast cancer [17–19]. Thus, the goal of this qualitative descriptive study was to elucidate the possible multi-level barriers—herein defined as range of constraint/determinants to early access to breast cancer diagnostic services among women occurring at various level of influence (e.g. the individual, family and social supports, local community environment, health system, and national environments [17–19]. The purpose is to inform the development of a multilevel intervention in breast cancer awareness and early detection in rural Tanzania, where the risk for late detection of breast cancer is higher compared to their urban counterparts [20]. The current study addressed the following specific research question guided by the socio-ecological model [3]: What are the reported challenges/barriers to breast cancer awareness and early screening among midlife women aged 40–65 years old in rural Tanzania? We focused on midlife women as one of the most common affected age group in Tanzania [3].

## Methods

### Qualitative approach and research paradigm

The Standards for Reporting Qualitative Research (SRQR) were used to report findings from this qualitative case descriptive study.

The study was a result of a partnership between the faculty at a local public University in Tanzania and a public University in the U.S. The study allowed an in-depth and comprehensive exploration of multi-level barriers to early detection of breast cancer among rural midlife women using multiple methods and samples [21]. The collection of data and presentation of findings were guided by the Socio-Ecological model. Being qualitative in nature, the study took an interpretative paradigm [21]. That is, we studied barriers to a social action [prevention of breast cancer] in a specific context (mid-life women in a rural setting) by interpreting meanings behind the identified barriers.

### Researcher characteristics and reflexivity

The interviews and focus group discussions were conducted by the first author who introduced himself as a public health faculty member with a background in nursing education from a Tanzania-based public university and an experienced researcher in conducting qualitative studies involving women’s health. Prior to data collection, I was concerned about how the male gender was going to affect targeted key stakeholders’ willingness to openly discuss the barriers to breast cancer awareness and early detection among mid-life women. However, this was not an issue after the introductions and relating with the male and female participants through shared experiences as a married man and public health professional.

### Study setting and context

This study was conducted in two villages in Ileje District, Tanzania between July and August 2021. The district is in Songwe Region, which is second to Dar Es Salaam with respect cancer burden Nationally. The Ileje district was selected because it is the most remote rural setting in Songwe Region. This is because such areas are characterized by low household income, low educational achievement, and weak health system infrastructure.

### Sampling strategy

The study employed a purposeful sampling strategy [22]. With assistance of Community leaders a total of 86 eligible participants (women aged 40–65 and their spouse) were identified, confirmed, and invited to participate in the study. However, 2 participants declined to participate in the study after being informed of what the study was all about. That is, the 2 did not consent. Thus, the data were collected from purposive samples [22] made of a) 10 *key informant interviews* (KII) with 5 health systems managers and 5 community health workers and b) 8 *focus group discussions (FDGs)*, (i.e., 3 groups with women only, 3 groups with men (spouses) only, and 2 couples-only groups), all of them with sum of 84 participants (see Table 1).

**Table 1:** Summary of sources and methods of data collection.

		Number of Participants by Village		
Data Source	Data Collection Method	Itumba	Isongole	Total
Health system managers	Key informant interviews	4	1	5
Community Health Workers	Key informant interviews	3	2	5
Women	3 FGDs	18	12	30
Men	3 FGDs	10	18	28
Couples	2 FGDs	4 couples (8 participants)	4 Couples (8 participants)	16
**Total**		**45**	**39**	**84**

These categories of respondents—health system managers, community health workers (CHWs), and community members (women and men) were purposefully selected because they are potential key stakeholders in developing and implementing community-based programs for cancer prevention in Tanzania. Specifically, the purpose was to gather information that will facilitate future interventions that will involve CHWs as key players to mobilize individuals, families, community and raise awareness about breast cancer in Tanzania. Recruitment of the respondents stopped after reaching data saturation—the point in data collection and analysis when new information produced little or no change to the progression of theme identification. Using a community engagement approach [23], local [village] government leaders and the Ileje District Medical Officer served as the community field guides. They were notified about the criteria for recruitment and helped to identify the eligible study participants. Then, two Tanzania-based bilingual interviewers (i.e. TN, the study investigator and a study research assistant) validated eligibility of the prospective participants and carried out data collection.

### Ethical issues pertaining to human subjects

The Muhimbili University of Health and Allied Sciences (MUHAS) Institutional Review Board approved and monitored this Wright State University-MUHAS collaborative study. The prospective interviewees and FGD participants were asked to take part in the study. The interviewer/moderator explained to the potential participants that their participation was entirely voluntary before they gave their consent. Additionally, they were told that no names were necessary and that the data would be treated with the utmost confidentiality.

As a result, the authors did not have access to information that could identify individual participants during or after data collection.

Additionally, participants were asked to maintain confidence by not disclosing to those who were not participants any personal experiences that might be discussed. Finally, the option to decline participation was explained to each prospective participant. Ultimately, they gave their written consent.

### Data collection methods

Data were collected using key informant interviews and focus group discussions (FGDs) that consisted of groups of 6–12 respondents. The data collection process was led by two facilitators: a moderator and a note taker (recorder). Participants completed a socio-demographic survey prior to the interviews and discussions.

### Data collection instruments and technologies

Data were collected using interview and focus group discussion guides which were structured around a set of open-ended questions that explored midlife women’s knowledge and experiences of breast cancer and breast examination as well as related barriers at multiple socio-ecological levels of influence (see Table 2 and S1 Appendix).

**Table 2:** Interview and focus group discussion guide sample questions, by content area.

Content	Example questions
A: General health problems and their solutions	• What are the main health problems facing women aged 40–65 years in this community (hamlet/village) • For each main problem mentioned, what health programs deal with the problem at the family, facility and community levels?
B: Breast cancer and breast self-examination to see if there is any lump:	• What experience do you have about breast cancer to women aged 40–65 years in this community (hamlet/village). • What are the benefits of cancer early detection among women in this community? • What are the obstacles on breast cancer early detection among women in this community? • What methods can women in this community use to detect breast cancer early? • What does the concept of breast self-examination mean to women in this community? • What are the challenges of doing breast self-examination at the family, facility and community levels?

### Units of study

A total 84 persons participated in the study (see Table 2)—of whom 10 were key informant interviews (health system managers and community health workers) and 74 were FGD participants whom 38 were women (see Table 1). Majority of the FGD participants were aged between 51–60 years (48.6%), had primary level of education (86.5%), and had monthly income of < USD 50 (78.5%) which is below the Anker Living Income reference for Rural Tanzania in 2020 of USD 200 per month [24]).

### Data processing

The data were gathered in Kiswahili, followed by verbatim transcription and translation into English (see S2 Appendix).

### Data analysis

The information in the transcripts was firstly unpacked and got organized in accordance to the levels of influence depicted by socio-ecological model—i.e individual factors, interpersonal factors, community factors, institutional factors, and societal factors. Then TN and RE independently reviewed and coded the transcripts using thematic analysis method [25]. The analysis was carried out in four stages [25, 26]: firstly, the line-by-line coding of field notes and transcripts; secondly, the in-depth examination and interpretation of the resultant codes and their categorization into descriptive themes; thirdly, synthesis of descriptive themes into more abstract analytical themes; and, fourthly, further condensation of analytical themes into the overarching theme.

### Techniques to enhance trustworthiness

Limiting cultural bias and enhancing trustworthiness of the results was achieved through several ways, such as bracketing [27], prolonged engagement, member checks and peer debriefing [28]. Bracketing was employed by discussing and being aware of self-assumptions and preconceptions about the topic during the design and implementation of the FGD as well as synthesis of the findings. Regarding prolonged engagement, the data collection period was planned such that the data collection team (TN and the Research Assistant) had time for reflection with RE between field visits and were therefore able to conduct preliminary analyses that guided their subsequent data collection. Moreover, a member check technique was applied during key informant interviews and FGDs whereby the interviewer/ FGD moderator summarized the information from the respondent/ discussant to ensure that what was heard was in fact correct. Additionally, peer debriefing was done during data collection and analysis whereby TN and RE independently reviewed and coded the transcripts, shared the outputs, discussed the disparities to ensure that final themes were grounded in the data. Finally, the paper benefited from triangulation [28, 29], as data from multiple methods (interviews and FGDs and sources (health systems providers, community health workers, women and their spouses) were synthesized to develop a comprehensive understanding of the possible barriers to breast cancer awareness and early detection.

## Results

The characteristics of study participants are shown in Table 3. The majority of the women and spouses (FGD participants) were aged 51–60 years old (48.6%), peasants (89.2%), had at least primary education (86.5%) and had a monthly income less than or equal to $50 (TZS 100,000) (78.4%). Other study participants (Key Informant interviewees) included 5 health systems managers (3 males and 2 females) at the district and health facility levels and 5 Community Health Workers (CHWs) (4 females and 1 male). On the education levels of the CHWs, 3 had primary level of education and only 2 had completed ordinary level secondary education.

**Table 3:** Sociodemographic characteristics of the FGD participants.

Characteristics		Frequency (n = 74)	Percentage
Sex	Female	38	51.4
	Male	36	49.6
Age	40–50 years	28	37.8
	60 years	36	48.6
	> 60 years	10	13.5
Occupation	Peasant	66	89.2
	Small scale business	4	5.4
	Peasant and Pastoralist	1	1.4
	Government employee	1	1.4
	Artisan	1	1.4
	Pastor	1	1.4
Education	Never been to school	2	2.7
	Primary education but did not complete	2	2.7
	Primary education	64	86.5
	Secondary education but did not complete	3	4.1
	Secondary education—Form IV	3	4.1
Monthly Income	_≤ 2TZS 100,000 (USD 50)	58	78.4
	> TZS 100,000 (USD 50)	16	21.6

### Barriers to early detection of breast cancer among mid-life women

Nine main [analytical] themes describing barriers to early detection methods among mid-life women were identified across five socioecological levels of influence (see Table 4 - illustration of thematic analysis process).

**Table 4:** How analytical themes emerged.

Level	Selected quote/ text	Codes	Descriptive Themes	Analytical Themes	Overarching Theme
**Individual Factors**	People in the villages face a lot of challenges but don’t know how to overcome them. As women, our response is very positive because we don’t want to suffer more consequences. Another thing is that, we just hear about breast cancer, a few days ago we heard that a student from Itumba Day [secondary school] had one of her breasts that was bigger than the other. She is still getting treatment (Woman, FGD, Itumba).	Rural dwellers, A lot of challenges, Don’t know how to overcome challenges, limited awareness about breast cancer, Just hear about breast cancer, Readiness of women, Suffer several social/ health problems Do not want suffer more consequences, Aware of women with breast problems	Rural women suffer from several social/ health problems Limited knowledge how to overcome challenges including breast cancer	Limited knowledge about breast cancer, including methods for early detection	Multi-level barriers to early detection of breast cancer among midlevel rural women in Tanzania
	*“We have no education*. *We do not know the causes of this disease [breast cancer] and its signs and its symptoms*. *Consequently*, *it is difficult to practice preventive measures*.*”*	Do not have education about breast cancer, Do not know causes of breast cancer, Do not know signs of breast cancer, No practice of preventive measures	No knowledge about breast cancer Inability to practice measures for early detection of breast cancer		
**Interpersonal Factors**	*We have no education about this disease*. *We men are not involved when it comes to intervention targeting women’s health problems*. *If we were knowledgeable we could have supported our spouses better*, *No man would want to see his spouse suffering*	Lack of knowledge about breast cancer Lack of men involvement Lack of knowledge constrains support Willingness of men to do something for the good of their wives’ breast health.	Men lack knowledge about breast cancer Men are not involved in interventions *targeting women’s health problems*, *including breast cancer*	Limited male partner support.	
**Community Factors**	*And our behavior*, *we Africans when we are not sick*, *we don’t go to the hospital*. *That I just leave home*, *I am physically fit and I decide to leave in the morning and go to the hospital and say “let them examine me” aah I have never seen anyone do that aah I have never seen*.	Behavioral, Africans, Do not go to hospital, Unlikely to leave home when apparently okay.	Seeking medical checkup is a behavioral issue It is not African culture African women are unlikely to bother themselves with seeking medical checkup when apparently okay	Procrastination	
**Institutional factors**	The screening services we have are for cervical cancer, not breast cancer. For cervical cancer, we conduct outreach programs. However, when a woman presents with a complaint about her breast(s) she is attended to accordingly.	Screening services for cervical cancer No screening services for breast cancer Outreach program for cervical cancer Complaint about a problem in the breast Services rendered as per the complaints presented	There are no routine screening services for breast cancer There is no outreach program for breast cancer Services for breast health are curative	Limited availability of breast cancer screening services and resources Predominance of curative care at the expense of preventive care	
**Societal Factors**	*Life is difficult*. *It is not easy for a poor woman to spend money on healthcare when she does not feel endangered*. *She does not go to hospital for routine checkup and our economy is contributing to this fate*. *When you see you just feel okay*, *you eat*, *you are safe*, *you can’t go*. *You just say God thank you*. *Because our rural economy is in shambles*, *deciding that I should be going for breast health checkup might be considered unnecessary expenditure*.	Life hardship, Poverty among women, Poor economic status *Unlikely to spend money for disease prevention* *on healthcare when she does not feel endangered* *Economic implications of breast checkup*.	Life hardship is a deterrent to practicing disease prevention A poor woman is u*nlikely to spend money for breast cancer screening when she does not feel endangered*	Poverty	

### Individual level factors

#### Limited knowledge about breast cancer and early detection

At the individual level, the majority of the women and their spouses indicated that they had heard of breast cancer. In addition, some participants mentioned that they knew someone with or suspected of living with breast cancer who had a successful or unsuccessful story related to early or delayed treatment. As one woman noted:

*People in the villages face many challenges but do not know how to overcome them*. *As women*, *our response is very positive because we do not want to suffer more consequences*. *Another thing is that*, *we just hear about breast cancer*, *a few days ago we heard that a student from Itumba Day [secondary school] had a painful breast that was bigger than the other*. *She is still getting treatment* (Woman, FGD participant, Itumba).

Many women attributed their awareness about breast cancer to a breast cancer awareness and screening campaign event that took place somewhat a while ago. In reference to the campaign, one woman stated, “*I learned about this disease when doctors from Dar es Salaam came to our district*. *It was a big campaign*. *We were informed about their presence at our district hospital*. *Many women went*, *they taught and examined us about breast cancer*.” (Woman, FGD participant, Isongole). This was also supported by one community health worker (CHW) who stated: “*More than five years ago*, *a group of experts came here*. *They were stationed at the District Hospital*, *and women from nearby villages went there for breast cancer investigations*.” (CHW, Isongole).

Despite reports on occasional breast cancer campaigns, all participants in the study admitted that limited knowledge was a major barrier to their uptake of breast cancer awareness and early detection strategies. As indicated by one of focus group discussion participants:

*“We have no education*. *We do not know the causes of this disease [breast cancer] and its signs and symptoms*. *Consequently*, *it is difficult to practice preventive measures*.*”* (Woman, FGD participant, Itumba).

CHWs and health care providers also presented lack of knowledge as a barrier. As one CHW puts it: *“lack of knowledge about breast cancer is a challenge*. *Most women are not aware of what they need to do to avoid breast cancer*.*” (CHW*, *Itumba)*. In terms of breast self-examination, the majority of women indicated a lack of knowledge and self-efficacy to complete the procedure: One FDG participant indicated: *“Most of us do not know and do not do breast self-examination*. *It could happen by chance when taking bath*, *one may notice something unusual in the breast; but deliberate attempts to do breast self-examination are almost nonexistent” (Woman*, *FGD participant*, *Isongole)*. Nevertheless, women knew that, if they noticed a change in their breasts, they should go to the hospital for further checkup and not to a traditional healer. Very few women and men were aware of mammography and its role in early diagnosis. Likewise, very few knew about clinical breast examination.

#### Witchcraft beliefs

Whereas the health care providers stressed the importance of early treatment in breast cancer control, beliefs such as witchcraft seemed to get in the way for some women, as expressed by one health care provider:

*According to my knowledge*, *I only know about three people who suffer from cancer in this village*. *After asking them*, *some of them associated it with superstitious belief but since we are experts*, *we knew that it was not caused by superstition so if they would have been educated*, *they would be treated in the health facility*. (CHW, Isongole).

### Interpersonal level factors

#### Limited male partner support

It was reported that support of the male partner in facilitating early diagnosis of breast cancer is limited, partly due to lack of knowledge about the disease. Male FGD participants attributed minimal knowledge to limited involvement of men in health campaigns. They cited an example of a breast health campaign that was carried about 5 or 7 years ago, [no one could remember the exact year]. One of the male FDG participant commented: *“We have no education about this disease*. *We men are not involved when it comes to intervention targeting women’s health problems*. *If we were knowledgeable*, *we could have supported our spouses better*. *No man would want to see his spouse suffer*.*” (Male FGD participant*, *Isongole)*.

### Community level factors

#### Age and gender norms

While most women stated that they had no preference regarding the gender of the CHW who would conduct their screening, some participants indicated a potential concern, especially with male and younger CHW. For instance, one male FDG participant shared the concern as follows:

*“It will be much easier for a female CHW especially if a woman is elderly*. *She might be an elderly woman aged 50*. *If a woman in her 50’s comes to me*, *she will not be comfortable but a woman with a woman/grandmother it is okay*. *She will feel shy when I ask her to undress so that I can examine her breast*. *The same applies to me if I go to the hospital and find a young lady asking me to take off my clothes*, *I won’t be comfortable*. *If you find a person of the same gender like yours*, *it is okay*. *There should be two genders so that if a person is not comfortable*, *they can just switch*.*” (Male FGD participant*, *Itumba)*

#### Procrastination

Procrastination of health screening was a major factor that hindered breast cancer screenings for participants. Female participants cited that women in the village did not have a culture of routine check-up, they did not need to see a doctor if they are not sick. That one would only go to a health facility when she feels unwell. One participant attributed this to low self-esteem (poor sense of self-value) as she said:

*We women have tendency of saying to ourselves I am apparently healthy why get bothered. We ignore going to health facility for a check up before experiencing sign of disease. So the constraint I am seeing is low self-esteem. This is what torments us, we women we lookdown upon ourselves. (Female FGD participant, Itumba)*

This was corroborated by male respondents who also pointed out that not accessing clinical breast exams is a cultural issue, that people in Africa seek healthcare when they are troubled. Likewise, “apparently healthy” African women are less likely to go to the health facility for breast check-up when there is no obvious outward swelling and there is no pain whatsoever. One FGD participant thus said:

*“And our behavior, we Africans when we are not sick, we don’t go to the hospital. That I just leave home, I am physically fit and I decide to leave in the morning and go to the hospital and say “let them examine me” aah I have never seen anyone do that aah I have never seen.” (Male FGD participant, Isongole)*.

This corroborated by another participant who asserted that Africans tend to neglect signs especially when the have low knowledge about the impending consequences:

*“I think it is lack of awareness, it is common for Africans not to take things seriously so whenever you see any sign you take it as a part of life. So, when the majority find something unusual, they take no action. They cannot do breast self-examination of the breast for something they do not know, even if has its negative effects. Only a few of them will see something unusual and seek medical attention. For the majority, one may ask herself ‘why do I feel something hard in my breast during bath’ but if she gets used to it, she will take it as normal.” (Male FGD participant, Isongole)*.

### Institutional level factors

#### Limited availability of breast health services and resources

FGD participants pointed out that they were aware of services for cervical screening at the district hospital, but not for breast cancer. This was confirmed by health system managers who admitted that educational and diagnostic services for breast cancer were limited. That the district did not have an organized way of delivering information about breast cancer. During this study, the district had a specific program for early detection of cervical cancer, which did little or nothing about breast cancer. As described by one of the key informants at the district level:

*“The screening services we have are for cervical cancer*, *not breast cancer*. *For cervical cancer*, *we conduct outreach programs*. *However*, *when a woman presents with a complaint about her breast(s) she is attended to accordingly*.” (Health System Manager, Itumba).

Moreover, the district health management confirmed that the district did not have mammography screening services, adding that such services can only be found at the National Hospital in Dar es Salaam, about a thousand kilometers away.

#### Predominance of curative care at the expense of preventive care

The managers of health facilities in the study area admitted that they were not aware of existence of the *National Guidelines for Early Diagnosis of Breast Cancer and Referral for Treatment* [14]. Moreover, there were reports on low priority to women’s breast cancer in the community, which constitutes a hinderance to breast cancer control at the dispensary and community levels. Although it is known that early detection of breast cancer saves lives, it was gathered that efforts towards that direction are minimal. All managers of health facilities admitted that they did not have the current *National Guidelines for Early Diagnosis of Breast Cancer and Referral for Treatment* [14] for breast cancer care. They further admitted that breast healthcare at the health facilities within the district is mainly curative, that there is no routine breast checkup for early detection of breast cancer. One of the managers succinctly commented:

*“We handle patients as per the complaints they come up with*. *If she does not report about abnormality in the breast*, *we do not examine it*.” (Dispensary In-charge)

Besides, both women and men who participated in this study reported that the health system does not encourage people to engage in preventive practices against breast cancer. One CHW cited that: “*We have heard from media that breast cancer exists*, *but people are not encouraged/interested to go for breast cancer examination*.*” (CHW*, *Isongole)*.

### Societal level factors

#### Poverty: Despair due to inability to pay

It was reported by both male and female FGD participants that women do not access clinical breast examination (CBE) because of despair associated with inability to pay. It is known that health services are not free and, because most women in the rural settings are poor, health checkup including CBE is not prioritized. The respondents argued that because of financial hardship women and most community members decide to seek healthcare and spend their meagre income when they fall sick:

*“Life is difficult. It is not easy for a poor woman to spend money on healthcare when she does not feel endangered. She does not go to hospital for routine checkup and our economy is contributing to this fate. When you see you just feel okay, you eat, you are safe, you can’t go. You just say God thank you. Because our rural economy is in shambles, deciding that I should be going for breast health checkup might be considered unnecessary expenditure.” (Male FGD participant, Isongole)*.

#### Limited health insurance scheme

It was reported by both male and female FGD participants that health insurance in the rural settings is failing to meet people’s expectations of accessing “free” services, thereby demotivating them from seeking healthcare including breast health checkup. Some participants argued that there is a mismatch between what people are told when they are being incentivized to become members of the Community Health Fund (CHF) and what they actually experience when they become members. They are told that if they enroll in the CHF, they would not incur extra costs when they fall sick. To the contrary, after they enroll, they realize the CHF does not cover all their health care needs. For instance, the respondents expressed their disappointment on out of stock prescriptions in the district hospital that they had to buy from private pharmacies. As one, the FGD participants stated:

*“It is true that people were very motivated to pay for insurance but even if you have insurance, you will often not get all the prescribed medicines. You get some and they tell you others are out of stock, you have to go and buy from the private pharmacy. The problem that I have seen is that when medicines are prescribed if they are 5, you will be given 2 and the rest you will be told to go and buy; and they even show you the shop/pharmacy. All these happen despite having health insurance.” (Male FGD participant, Isongole)*.

## Discussion

Overall, the study findings provide a better understanding of individual-level determinants for knowledge deficits about breast cancer awareness and early detection within a broader environmental context. For example, at the individual level, the findings on low risk perception about breast cancer align with previous studies conducted in the region [30], suggesting the importance to raise awareness regarding the risk and protective factors for breast cancer among women, especially in communities with low level of educational achievement, as evidenced by the majority of FGD participants (86.5%) in this study who had primary level of education. At the interpersonal level, the findings are corroborated by similar studies in other countries such as Ghana [31] and Saudi Arabia [32] on the importance of male partners’ [spouses’] involvement. Moreover, the participant’s culture of complacency on routine checkup seemed to be attributed to community level and institutional/systems/organization-level breast cancer-related health-seeking behaviors such as: 1) sheer negligence, 2) women’s low risk perception due to limited or lack of knowledge and 3) nurturance by a less responsive health system characterized by long waiting times and payment requirements. This finding is consistent with findings conducted in Wakiso, Uganda [33].

Other determinants at the institutional level include the lack of educational services, routine clinical breast examination and continued access to a predominantly curative health care system. These factors have also been reported elsewhere by Philipo et al. [6]. The limited availability of institutional-based services for early diagnosis of breast cancer might be attributed to the delays in implementation of the recently approved *National Guidelines for Early Diagnosis of Breast Cancer and Referral for Treatment* [14]. Chao et al. [2] have reported comparable findings in the Northern region of Tanzania. At the societal/policy level, despair associated with inability to pay was identified as a factor that influenced the uptake of clinic-based clinical breast examination. This finding has also been reported in other studies in 15 low-income countries (Chad, Mali, Congo, Comoros, Laos, Zimbabwe, Burkina Faso, Nepal, Mauritania, Myanmar, Ghana, Kenya, Malawi, Ethiopia and Bangladesh) [34, 35]. Moreover, the health insurance schemes, such as iCHF and NHIF, in Tanzania, that take a curative approach in their coverage of expenses related to management of non-communicable diseases including breast cancer [34], continue to imply challenges related to preventive health care financing. [36, 37].

### Limitations

First, the scope was limited to mid-life rural women, and second, the nature of the purposive sampling approach may provide missed opportunities for inclusive perspectives from community members and health care providers. Nonetheless, this is the first study that we are aware of that has collected and triangulated data from women and their spouses as well as health care providers to understand the multilevel barriers and facilitators of breast cancer awareness and early screening prior to the development of a multilevel intervention in the region.

### Generalizability

This being a qualitative study, its goal was not to generalize but rather to provide a rich, contextualized understanding of barriers to early detection of breast cancer. Nevertheless, in the qualitative perspective, the study has achieved both analytic generazability and transferability (reader generalizability) [38]. Regarding analytic generalizability, the authors developed conceptualizations of mid-life women’s experiences through in-depth scrutiny and higher-order abstraction into themes. Moreover, on transferability, they have provided detailed descriptions that will allow readers to make inferences about applying the findings to other similar populations and settings.

## Conclusion

This study has explored multi-level barriers to early detection of breast cancer among midlife women in Tanzania. They include: limited knowledge about breast cancer and early detection; witchcraft beliefs; limited male partner support; procrastination; limited availability of breast health services and resources; predominance of curative care at the expense of preventive care; despair due to inability to pay (poverty); and limited health insurance scheme. These study findings contribute to the accumulated exciting empirical support for the need to further the design, implementation and evaluation of evidence-based community breast health awareness and education interventions to promote early detection of breast cancer. Specifically, the study highlights the need to address the identified multiple level determinants of influence as part of the country’s community health measures to mitigate the prevalence of delayed presentation of breast cancer among women in rural settings.

## Supporting information

S1 Appendix FGD guide. (DOCX)

S2 Appendix FGD transcript. (DOCX)
