# Supplementary material for: Multi-level barriers to early detection of breast cancer among rural midlife women in Tanzania: A qualitative case study
Source: PLoS One. 2024 Feb 29;19(2):e0297798. doi: 10.1371/journal.pone.0297798 (PMC10903879; doi:10.1371/journal.pone.0297798)
Supplement: S1 Appendix — (DOCX) [file pone.0297798.s001.docx]

**S1 Appendix**

**SAMPLE FOCUS GROUP DISCUSSION GUIDE**

**FOR USE WITH WOMEN/PARTNERS/ MARRIED COUPLES ON THE DEVELOPMENT OF AN AWARENESS AND EARLY DETECTION PROGRAM OF BREAST CANCER AMONG WOMEN AGED 40 - 65 IN THE DISTRICT OF ILEJE, TANZANIA**

**Introduction: Good afternoon? My name is** *[mention the moderator’s name] and I'm working on a breast cancer research project. Thank you for coming. Today we are here to learn about your life especially you adults living in Ileje District. We need to talk about Your Life about* ***awareness and beliefs*** *about breast cancer,* ***access*** *to breast health care services, personal breast care* ***practices****, and breast examination* ***services*** *at health facilities. We want to know about the existing information on breast cancer. Before we get started, we will provide you with more information about what our discussions will be like, so that you can decide whether you can participate or not.*

**Read the Consent Form**

**Take personal information of each participant [Use an attached form for each participant]. Interview guide for members who will participate:**

- *This is an informal discussion, so do not wait until I mention you by name. In fact, I ask each participant be free to contribute as he or she follows other participants’ comments.*
- *If you do not understand the question, please let me know.*
- *I request every participant to keep this conversation confidential; do not mention the names and opinions of the participants anywhere after leaving here. I believe you will feel free to speak openly and truthfully.*
- *Please speak one after another.*
- *To ensure that we move forward, I can interrupt to make sure we discuss all the questions.*
- *There is no right or wrong answer.*
- *Disagreements between participants are allowed*
- *Each person should try to participate/share his/her opinion.*
- *Follow the discussion and please do not have side conversations.*
- *This discussion will be recorded for the purposes of this study only.*
- *Apart from top officials in the study, no one else will listen to the conversation, which will be recorded, and after the study is completed this audio files will be deleted.*

Date of interview: ……………………………………………………

Place of interview: ……………………………………………..

Moderator: …………………………………………………………….

**Verify participants' understanding of the discussion:**

*Is there any person who has a question or needs further clarification before we begin?*

**The discussion begins: [Switch on your recorder**.]

**A: General health problems and health service programs**

***Let us begin by talking* about general health problems and existing health service programs in order to solve those problems.**

1. What are the main health problems affecting women aged between 40 - 65 in this community (hamlet/village):
2. Probe further: cancer?
3. Probe further: others?
4. What is your opinion on providers for those main health problems? Use the following probes for each problem mentioned:
5. Probe further: Individual/family efforts– example partners?
6. Probe further: Efforts at community level?
7. Probe further: services at the health facility.
8. For each mentioned main problem above, probe further about the existing health program (how that program works):
9. Probe further: Program at individual/family (example partners)?
10. Probe further: Program at community level?
11. Probe further: Program at the health facility.

**B: Breast cancer awareness, breast self-examination, Breast examination performed by health specialists:**

**Our intention is to know your understanding on breast cancer, breast self-examination, and Breast examination performed by health professionals to women aged 40 – 65 in this community (hamlet/village).**

1. Let us talk in details about breast cancer for women aged 40 – 65 in this community (hamlet/village).
2. Probe further: what do you understand about breast cancer?
   - Perceptions of people here about causative agent [causes]/ things which put more risk of this disease
   - Perceptions of people here about preventive measures against this disease
3. Probe further: On average, how many people suffer from this disease? {few / many)
4. Probe further: How dangerous is this disease?
5. Probe further: To what extent women are at risk of getting this disease?
6. Now, let us talk about the benefits of early detection of cancer among women in this community.
   1. Probe further: What are the benefits of cancer early detection among women in this community?
7. Let us discuss different methods that women in this community can use to detect breast cancer early.
   1. Probe further: What methods can women use to detect breast cancer early in this community?
8. Now, let us discuss different methods that women use to detect breast cancer early. [Show them you are aware if the method has been mentioned in question 6a]
9. About breast self-examination:
10. Probe further: what is your opinion about breast self-examination
11. Probe further: how many women do you think perform breast self-examination? (many/ few?)
12. Probe further: how often do you think women in this community perform breast self-examination?
13. Probe further: do you think women in this community are carefully in performing breast self-examination?
14. Probe further: where do women perform breast self-examination in this community?
15. What are the challenges of performing breast self-examination?
16. Probe further: challenges at individual level, family level, community level, cultural level, systems levels/health facilities government and private level.
17. About the examination done at the health facilities
18. Probe further: what is your opinion about examination done at health facilities?

iii. Awareness about examination performed at health facilities

1. Probe further: The number of women who seek for breast examination services at the health facility in this area
2. Probe further: How often do women access breast cancer examination service at the health facility in this area
3. Probe further: do you think women in this community are carefully in seeking for breast cancer examination at the health facilities in this area?
4. Probe further: where do they normally go for breast cancer examination performed by a health specialist
5. Probe further: what are the challenges of accessing breast cancer examination services at the health facilities?
6. Probe further: Individual level, Family level? Community level? Health systems levels- public and private? Cultural level?
7. Now, let us talk about challenges that affect women from early detection of breast cancer (early stages of the disease).
8. Probe further: Apart from the ones you have mentioned, what are other challenges affect women from early detection of breast cancer (when the disease is at early stages)?
9. Now, let us talk about actions that can be taken in order to enable women to detect breast cancer early when it is still at early stages.
10. Probe further: In general, what can women do in this village in order to detect cancer early, including obstacles/existing challenges when it is still at early stages? Or if they mentioned challenges,
11. Probe further: what can women do in this village in order to detect cancer early when it is in early stage, including the challenges that have been mentioned?
