# Supplementary material for: Multi-level barriers to early detection of breast cancer among rural midlife women in Tanzania: A qualitative case study
Source: PLoS One. 2024 Feb 29;19(2):e0297798. doi: 10.1371/journal.pone.0297798 (PMC10903879; doi:10.1371/journal.pone.0297798)
Supplement: S2 Appendix — (DOCX) [file pone.0297798.s002.docx]

**S2 Appendix**

**SAMPLE FOCUS GROUP DISCUSSSION**

**SPOUSE (MALE) TRANSCRIPT**

**FGD 7: WANAUME/MEN – [LOCATION MASKED FOR CONFIDENTIALITY].**

M: Sawa. Basi kama mko vizuri tuanze. Swali la kwanza ambalo tunataka kuanza nalo ni kwamba hapa katika Kitongoji cha [masked for confidentiality] mnapozungumza mnafikiria [masked for confidentiality] lakini mnaweza mkafikiria mpaka kijiji cha [masked for confidentiality] kata au wilaya ya Ileje. Kwamba matatizo makubwa ya kiafya yanayowapata kina mama ni yapi? Matatizo makubwa ya kiafya hasa magonjwa makubwa yanayowapata wamama wa hapa?. Ndio maana tuliwachagua ninyi kama wanaume ambao mko kwenye ndoa ili mnaposema mnatoa maoni ya kifamiliya. Haya twende matatizo makubwa ya kiafya namba 2.

**Okay. So if you are good let's start. The first question we want to start with is that here at** [masked for confidentiality] **Harmlet when you speak think of** [masked for confidentiality] **harmlet, but you can think of the village of** [masked for confidentiality]**, the Ward or Ileje district at large. What are the major health problems facing women here? Serious illnesses that afflict local mothers here? That is why we recruited you as married men so that when you speak, you provide the perspective of the family. Let's start, serious health problems, number 2.**

Recorder: namba 2/ number 2

P2: Matatizo makubwa ninayoyaona siku hizi kwa kina mama wengi ni matatizo ya miguu

**The biggest problems I see these days for most mothers are foot problems.**

M: Matatizo ya miguu. Miguu inakuwaje?.

**Foot problems. How are the feet/ how do the problems manifest?**

P2: Unakuta humu wengine inavimba alafu unakuta anatembea kwa shida asilimia kubwa ambao ninawaona ni kina mama.

**You find some experience swelling and some walk with difficulty. A large percentage of these are women.**

M: Basi nimesikia tatizo, akina mama wanaumwa miguu, wakina baba kama nyie kama je miguu haiwasumbui? matatizo ya miguu sio mengi sana?

**So, I have heard the problem being said, women experience feet ache. Fathers, like you, don’t they experience feet problems?, so the problems of the feet are not so great?**

P2: Aah sijayaona mimi kwa sasa hivi labda wenzangu.

**Aah I haven't seen them, maybe my colleagues**

M: Ni kwa kina mama zaidi. Asante, karibu mwingine, tatizo lingine la kiafya.

**It is mostly for women. Thank you. Welcome, another respondent, another health problem?**

Recorder Namba 5 / **Number 5**

P5: Tatizo ninaloliona kwa kina mama sana ni kifua

**The most troubling problem that I see in women is the chest illness**

M: Kifua

**The chest**

P5: Wanajisikia uzito sana kifuani.

**They feel very heavy in the chest.**

M: Kifua kinabana?

**Chest tightness?**

P5: Eeh. Yani utakuta wanalalamika tu kifua, kifua

**Eeh. That is, you will find them complaining about the chest, chest**

M: Anhaa asante. Mwingine zaidi ya kifua. Vipi maswala ya saratani. Kansa kwa kina mama. Karibu namba 7.

**Thank you. Other than the chest, how about cancer issues. Cancer in women. Welcome number 7.**

Recorder: Namba 7/ **Number 7**

P7: Asante nashukuru. Tatizo la saratani kwa wanawake lipo. Kwa sababu kuna baadhi ya wengine mpaka mwaka jana tumezika kwa sababu ya kansa. Licha ya wamepata operasheni ya matiti wamezunguka wameenda mpaka Malawi wakatibiwa ikashindikana kwa hiyo matatizo ya kansa yapo wakaenda hadi Mbeya ikashindikana.

**Thank you. The problem of cancer in women exists, because some died last year because of cancer. Despite having had breast surgery, they went around and went up to Malawi got treated but in vain. So, the cancer problems are evident. They went up to Mbeya but did not get cured.**

M: Kansa ya nini?

**Which type of cancer?**

P7: Kansa ya matiti nayo ipo.

**Breast cancer exists**

M: Anhaa. Kansa ya matiti ni kubwa kiasi gani, wamama wengi wanaugua kansa au wachache.

**Anhaa. How big is the problem of breast cancer, most mothers suffer from cancer or only few.**

P7: Wanakua nayo. Idadi angalau inaelekea elekea kwa sababu kwa maeneo ya kwetu inatokea nadra sana maswala ya uchunguzi.

**They have it (Breast Cancer), although it is difficult to establish the exact number because in our areas it is very rare to have screening.**

M: Maswala ya uchunguzi. Yaani uchunguzi wa kuangalia matiti ya mama inakuwa kwa nadra.

**Screening issues. That is, you are saying screening for breast cancer is rare**

P7: Kwa sababu ilipaswa vijana wa miaka 18 wafanyiwe uchunguzi kwasababu na wao wanashambuliwa na tatizo la kansa.

**Youth 18-year-olds ought to be screened as well because they too do suffer from cancer.**

M: Kansa. wako hata vijana ambao wana shida ya saratani ambao mnawafahamu?.

**Cancer. There are even young people with cancer who you know?**

P7: Eeeh miaka 18 wanakuwepo maana unakuta binti yupo shuleni unaweza ukasikia anasema anaumwa matiti. Sasa hiyo ni dalili ya kuwa msaada serikali ingetoa msaada uchunguzi ufanyike hata mashuleni.

**Eeeh 18 years old, they are there because you find a girl at school you can hear her saying she has a breast problem. Now that is an indication that government support is needed, to provide support screenings out in schools.**

M: Sawa asante. Mwingine.

**Ok. Thank you. Another participant?**

Recorder: Mwingine namba 3/ **Another participant?, number 3**

P3: Wanawake kutokutoa hedhi.

**Women not menstruating.**

M: Wanawake kutokutoa hedhi!

**Women not menstruating!**

P3: Eeh yani mpangilio wao haupo kamili.

**Eeh, that is, the order is not perfect (irregular menstruation)**

M: Mpangilio usio kamili na lenyewe ni tatizo

**Irregular menstruation is a problem**

P3: Eehe/ **Yes**

M: Aya asante sana.

**Ok. Thank you very much.**

Recorder. Namba 4/ Number 4

P4: Tatitzo lingine linalowakuta kina mama utakuta wakati mwingine ana mimba tatizo ambalo linawachanganya sana ni kule kupewa operasheni mara kwa mara na kuwa hospitali wanakuwa wengi sana. Kwa hiyo hili tatizo hatuligundui kuwa ni tatizo gani?

**Another problem that mothers face is that when they are pregnant they give birth by operation (C-section). There are several women who give birth (deliver) by cesarean section (operation); the number is increasing steadily. We do not know what could be contributing to this trend.**

M: Operasheni za mara kwa mara za uzazi. Kwa kujifungua kwa njia ya operasheni. Kwamba zimekua nyingi wengi wanaoenda kujifungua wanajifungua kwa operasheni.

**Frequent Cesarean Operations. That many women who go to a health facility for delivery go through a Cesarean Operation,**

P4: Eeh/ Yes

M: Kwa hiyo mnaliona kama tatizo.

**So you are seeing it as a problem.**

P4: Ndio/ **Yes**

M: Anhaa. Sawa sasa niulize habari ya huduma, upatikanaji wa huduma kwa magonjwa ambayo mmeyataja. Matatizo haya huduma zake zipo? Kwa mfano mmezungumzia hiyo miguu, huduma zake zipo? Wakienda kwenye vituo vya afya kwa mfano kwenye zahanati matibabu ya hiyo shida ya miguu, mnaweza mkazungumzia chochote katika hayo mliyoyataja. Kuna miguu, saratani, mmezungumzia hedhi, huduma wanazipata?

**Anhaa. Okay now let me ask about the care, access to care for the diseases you mentioned. Are the services for these problems available? For example you mentioned feet problems, are their services available? If they go to health facilities for example at the clinic for the treatment of the foot problem, you can talk about any of the things you mentioned. You have pointed out legs, cancer, and menstruation. Do people get the services?**

Recorder: Namba 1./ Number 1

M: Karibu Namba 1

**Welcome number 1**

P1: Ni kwamba huduma nyingi hazipatikani hapa ndo maana tunaenda Malawi.

**Most services are not available here, that is why we go to Malawi**

M: Ndo maana mnaenda Malawi

**That is why you go to Malawi**

P1: Eeeh. Kwa kwetu huku huduma ni hafifu sana.

**Yes, this side of ours, services are very poor**

M: Kwa nini mnaenda Malawi wakati kuna hospitali ya Wilaya na hapa ipo Zahanati?. Kwani kwenye Zahanati ya hapa [masked for confidentiality] hakuna huduma?

**Why are you going to Malawi when there is a District Hospital and here there a dispensary? Are services not available at the** [masked for confidentiality] **dispensary?**

P1: Huduma zinaweza zikawepo lakini mara nyingi huwa tunaambiwa tukanunue dawa. Kwa hiyo kwa ajili ya ukosefu wa pesa tunaenda Malawi.

**Services may be available but we are often told to buy medicines. Therefore, for lack of money we go to Malawi.**

M: Ukosefu wa ela.

**Lack of money**

P1: Eeh kule tunatibiwa bure.

**Eeh there we are treated for free.**

M: Kule Malawi mtatibiwa bure. Hata mkiambiwa muende Hospitali ya Wilaya Itumba watu hawaendi sana kwa sababu ya ela?

**In Malawi, you get treated for free. Even if you are told to go to Itumba people do not prefer going there because of lack of money?**

P1: Eeh/ Yes

M: Si kuna bima, kwa nini wasitumie bima. Wakate bima alafu watumie bima.

**Isn’t there health insurance, why don’t they use health insurance? Let them pay health insurance premium and then use the insurance.**

P1: Ni wachache wenye bima.

**Few are insured.**

M: Wachache wenye bima. Kwa nini wachache wana bima.

**Few are insured. Why only few are insured?**

Recorder: Namba 2./ **Number 2**

P2: Ni kweli watu walihamasika sana kukata bima lakini hata ukiwa na bima tatizo ambalo nimeliona mimi, unafika pale lakini ambae hana bima anahudumiwa haraka kwa sababu yeye anatoa cash pale pale. Lakini mwenye bima utazungushwa utaaidiwa lini unamuona daktari, dawa utaandikiwa mbili zingine utaambiwa hazipo kama zipo tano zingine wanakwambia utaenda kununua tena wanakuonesha na dula la kwenda kununua. Lakini una bima. Unapokata bima unamaanisha mimi najihami kwa sababu sina kipato nijikusanye ili siku ninapopatwa na imejensi nipate nini matibabu. Sasa wanakwambia wewe uliyekata bima duka lile pale kanunue dawa. Sasa sijui nani anawaambia kwamba duka lile ndo lina dawa.

**It is true that people were very motivated to pay for health insurance but even if you have insurance the problem that I have seen, you get there but the uninsured is quickly served because he pays cash right there. However, the insured person will not be attended to immediately, and you will be given an appointment when to see a doctor. The medicine will be prescribed, if they are 5, you will be given 2 and the rest you will told to go and buy; and they even show you the shop/ pharmacy. All these happen despite having health insurance. When you pay for health insurance it is like you are practicing self-defense in advance. You are essentially saying since I am a low income person let me pay health insurance premium so that when I fall sick (when I get an emergency of sickness) I can get treatment. Now they tell you who is insured “there is the shop, go and buy medicine”. I do not know who tells them the shop where to access the medicine.**

M: Sawa. Asante

Ok. Thank you

Recorder: Namba 7/ Number 7

P7: Asante nashukuru ni kweli tatizo kama hili la miguu hata kama ungeweza kupata matibabu kwa huduma ya huku kwa miguu ni ngumu sana, haipatikani. Kama walivyosema wenzangu, hata kama una bima. Huko nyuma tulikua na bima ya shilingi elfu 10 ambayo ulikua unaruhusiwa kutumia ndani ya halmashauri ya Ileje lakini saivi imekuja bima ambayo unatoa shilingi elfu 30 kwa familia mnaweza mkaungana kama watu 6 yani ya inahudumia kwa ajili ya mkoa mzima. Na ukienda pale unaambiwa kwamba kanunue dawa hata kama unakua na bima unaambiwa ukanunue dawa. Sasa unakuta unajiuliza kwamba je lile duka lina nguvu lina kipato kuliko serikali? Mimi ninaweza kuwa na duka langu binafsi la dawa lakini haliwezi likaizidi serikali hadi wagonjwa wanalazimika kupata dawa nje ya Hospitali. Lakini inawezekana miongoni mwa wahudumu au watumishi wanaweza wakawa na duka wakachukua dawa zikaingia pale kwenye duka la private maana. Kwa nini mtu akwambie kanunue dawa kwenye duka lile pale, ameonaje pale kwa nini serikali isihudumie wananchi kwa sababu wananchi wanalipa kodi, wanatoa michango mbali mbali alafu anakua na bima ile ela ya bima kwa nini isiende ikachukua zile dawa kwa hiyo ni tatizo ambalo linafika mahala wananchi kwamba basi potelea mbali. Kwa sababu ukienda Malawi hata kama utakufa wao wanakusafirisha bure wao hakuna gharama hata unayotoa. Wanakusafirisha bure mpaka kukuleta huku.

**Thank you, the foot ache it is really a problem here. Even if you could get treatment, treatment of foot problems, is difficult, it is not available. As my colleagues have said, even if you have insurance; in the past we had insurance of 10 thousand shillings which you were allowed to use within Ileje council but nowadays one is required to pay health insurance premium worth 30,000 (TZS) which caters for 6 people in the family, it enables access to services in the whole region. In addition, if you go there you are told to buy medicine even if you have health insurance. Now you ask yourself is medical store stronger than the government? I may have my own private shop but it can't exceed the government’s in stocking medicines. But it is possible that among the attendants or staff they may have a shop and take the medicines from the Hospital pharmacy into their private medical store. Why should someone tell you to buy medicine from a private medical store, how has he come to know that place? why doesn’t the government serve the people? Because the people pay taxes, pay various contributions and then they have health insurance. Why doesn’t the government use health insurance money to procure adequate medicines? Such situations cause people to lose hope and decide to go to Malawi. Because if you go to Malawi even if you die they transport your remains back free of charge. They transport you free of charge.**

M: Yani unasema mtanzania akitoka kama nyie akienda Malawi akaugua bahati mbaya akafariki wa Malawi wanamsafirisha kumtoa kule.

**That is, you are saying if a Tanzanian goes to Malawi and falls sick and unfortunately dies, the Malawians will transport the body back home.**

**P7**: Wao wanamleta moja kwa moja bila gharama yoyote.

**They bring him directly at no cost.**

M: Hapa Tanzania?

**Here in Tanzania**

P7: Eeh. Tunampokea, wanamleta hadi ofisi za uhamiaji za hapa kwetu, Wanatoa taarifa amekufa kwa sababu gani kwa sababu anakua na vyeti vya matibabu.

**Yes. We receive him here, they bring him to the immigration offices here, They inform them [Immigration] why he died because they come with medical certificates.**

M: Anhaa kwa hiyo wanamleta. Kwa hiyo serikali ya kule inahusika kumsafirisha kwa usafiri wa serikali.

**Anhaa so they bring him. Therefore, the Malawian government is responsible for transporting the body.**

P7: Serikali ya kule ndo inamsafirisha mpaka huku [masked for confidentiality], hamna gharama yoyote ni bure tu, ndicho kinachotusaidia.

**The Malawian government is transporting him to** [masked for confidentiality]**, at no cost, it is just free, that's what helps us.**

M: Hiyo tena sijawahi kusikia.

**I have never heard of that.**

Recorder: Anataka kuchangia namba 6/ Number 6 wants to contribute

P6: Mimi swali langu linalotupa sana kero huku kwetu ni juu ya kina mama miguu inawauma sana. Sasa tunashindwa kuelewa hata ukienda hospitali kama una bima anakupa dawa chache nyingine anakwambia nenda kanunue pale sasa aliyeweka ni nani? Lakini nimechangia shilingi ngapi elfu 30. Anakwambia nenda kachukue pale. Sasa maana ya bima maana yake nini. Inakua kero kwetu.

**My question that bothers us the most is about women. Their feet hurts a lot. Now we fail to understand even if you go to the hospital and you have insurance they give you few medicines. He then tells you go buy other medicine there, we wonder who stocks the pharmacy! I have contributed 30,000 shillings (for health insurance). But I am still asked to go and get the medicine at the local pharmacy. Now what does insurance mean? It bothers us a lot.**

M: Hiyo ni kero kweli, najua.

**That’s a real nuisance, I know.**

P6: Inatupa shida sana hiyo- unaumwa umetoa bima anakwambia nenda kachukue pale dawa.

**It gives us so much trouble - you are sick you have submitted health insurance card yet he tells you to go and get medicine there [at the local private pharmancy].**

M: Ni kero kwa watu wengi. Tutafikisha hiki kilio.

**It is a nuisance to many people. We will convey/share your concerns**

M: Karibu/ Welcome

P: Lingine mimi nilikua nafikiria kwamba sijui hii miguu ya kina mama ni kwa sababu wengine wanavyotumia sindano za majira au vidonge au tatizo gani?

**Another reason that I think could be contributing to women’s foot problems, I don't know, probably it is because of them using injectable contraceptives or pills or another problem?**

M: Ni tatizo la kufanyia utafiti hatuwezi tukasema haraka haraka.

**It is a problem that needs research, we cannot say confirm the cause without evidence.**

P: Kwa sababu wanaume asilimia kubwa unakuta husikii miguu ikiuma ukisikia anasema miguu ni wanaume ambao sasa umri umekwenda. Lakini akina mama unakuta labda ana uzao mmoja ameshaanza kutumia hata vidonge mwingine ametumia sindano za majira ameolewa mwaka huu anasema anataka azae baada ya miaka mitano mwaka huo huo unakuta tayari. Inawezekana hata mambo ya namna hii vinatumika. Labda aangalie na dawa yake za uzazi wa mpango, je vinaoana mwili wake? kwa sababu unakuta wengine hawatumii na hawana matatizo ya miguu.

**Most men do not complain about legs ache. When you hear them complaining, they are older men. But for women you find maybe she has one offspring (child) and she has started taking contraceptive pills, she has used injectable contraceptives. She is married this year and she says she wants to give birth after five years. It is possible that even things like those contribute to the problem. Maybe she should check the contraceptive pills she is taking, are they compatible with her body? Some women who not use contraceptives do not have feet problems.**

M: Vyote vinaweza vikachangia inategemea na mazingira.

**All of these can contribute, it depends on the circumstances.**

Recorder: Labda baadae nitaelezea tukimaliza kipindi ninavyofikiri ngoja tuendelee kidogo.

**May be I will explain after finishing the session, let us proceed a little bit.**

M: Sawa asante sasa tuendelee. Nilikua nazungumza huduma ndo tumefika hapo mmezungumza mambo ya bima. Tuendelee sasa baadae nitauliza huduma juu ya saratani ya matiti lakini kwanza tuzungumzie kuhusu uelewa wa wananchi nyie wenyewe mkisikia hili neno kansa ya matiti au saratani ya matiti mnaelewa nini na mkizungumzia watu wanavyozungumzia kwa ujumla tukianzia na nyie wenyewe. Mtu akiwauliza kansa ya matiti ni nini mnaelewaje. Mmetuambia kwamba hili tatizo lipo eeh watu wanaumwa wanaenda Malawi na nini kwa ajili ya kupata huduma. Sasa mtu akiwauliza saratani ya matiti ni nini kwa mujibu mnavyoelewa nyie kwa jinsi mnavyofahamu.

**Okay thank you now let's move on. I was talking about services and you introduced health insurance issues. Let's move on, now I will ask you questions about breast cancer but first let's talk about the understanding of community members whom you are representing, when you hear this word breast cancer what do you understand, talk about what people perceive in general, starting with you. When someone asks you about breast cancer, what do you understand? You have told us that this problem exists eeh people who fall sick and go to Malawi for accessing care. Now if someone asks you what breast cancer is, according to your understanding, what would you say?**

Recorder: Namba 2/ Number 2

P2: Kwa jinsi mimi ninavyofahamu sio kwa kufahamu tu ila kwa kuona. Nilikua nasikia tu vyombo vya habari saratani ya matiti lakini mwaka jana tumezika shemeji yangu amesumbuliwa sana na matiti kwa miaka 10, wakamkata lile titi wakaenda Dar es salaam mpaka Mzuzu .

**As far as I know, it is not just by knowing but also by seeing. I used to just hear about breast cancer but last year we buried my sister-in-law who was very much troubled with the breast for 10 years ago, they cut off. She went to Dar es Salaam, up to Mzuzu for care.**

M: Malawi?

P2: Eeh Malawi lakini baada ya kurudi akakaa akawa analima vizuri tu baada ya miaka kama 2 tena lile tatizo likaibuka tena. Sasa kuibuka kule kwa sababu ni shemeji yangu mimi nilikuwa namwangalia yaani unakuta titi hapa pamechimbika mpaka hata mfupa mule ndani inaonekana. Kwa hiyo ndipo nikagundua kwamba alaaa kumbe saratani ya matiti ndiyo ilivyo na ubaya kiasi hiko, kwa kuona sio kwa kusikia.

**Yes, Malawi. But after returning she settled down and became a good farmer for about 2 years, after which the problem arose again. Now after the cancer relapsed, because she is my sister-in-law, I took care of her. I could see the deep ulcer and the bone through her breast. So then realized, firsthand, that breast cancer is the worst thing that can happen to a person.**

M: Mmmm kweli kweli. Asante. Mwingine saratani ya matiti ni nini

**Mmm true, true. Thank you. Another participant? What is breast cancer?**

Recorder: Namba 7/ **Number 7**

P7: Asante. Mi nadhani ni kweli inawezekana kuna mdudu anakuwepo kwenye titi kwa sababu kama ni titi linaanza bila tatizo lolote nadhani inawezekana kuna chanzo ambayo inasababisha saratani iweze kuingia kwenye titi. Mi nadhani kuna chanzo ambacho tunashindwa kugundua tatizo gani. Inawezekana kuna mdudu ambae anaweza kupekenya titi alafu anaingia kwenye mfupa wa titi alafu baadae inajitokeza imeota. Kama alivyosema inajitokeza inasababisha vidonda. Unakuta mtu mahali pale pale panachimbika panaoza hata kama ingekuwa nini. Nafikiri inawezekana kuna mdudu Fulani labda mtatusaidia ninyi mnaoelewa.

**Thank you. I think possibly, there is a bug in the breast, because if the breast has no problem/issue there must be a source/agent that can cause cancer to enter the breast. I think there is a source that we have failed to discover. It is possible that there is a bug (worm) that can scratch the breast and then enter the breastbone and then later appearing as a swelling from within the breast. As he said, it turns out later that the bug can cause ulcers. You find that the deep ulcerated wound decays. You who are the experts, you can help us understand.**

M: Eeh. Asante baadae mwishoni tutawaambia. Sasa nataka kujua bahati nzuri sio nzuri mbaya yani hilo tatizo mmeliona hapa. Je watu wengi hapa wanajua hilo tatizo lipo hapa kijijini katika eneo hili la [masked for confidentiality] enhee wao wakiona mtu ameumwa, hivyo ameenda Mzuzu, ameenda Mbeya rufaa wanachukuliaje? Wanaelewa kwamba ni saratani ya matiti au wanakua na imani tofauti labda sio saratani ni kitu kingine na wanadhani nini kinasababisha. Nataka mtuambie mitizamo ya watu kuhusu huu ugonjwa.

**Eeh. Thank you, later at the end we will tell you. Now, I want to know, fortunately or unfortunately you have experienced that problem here, and if many people here know that the problem exists in this village of** [masked for confidentiality]. **Enhee when they see someone is sick so she has gone to Mbeya Referral Hospital, how do they understand the problem of breast cancer or they have different beliefs like they may say maybe it is not cancer it is something else, what do they think might be the cause? Else and they think what is causing it. I want you to tell us about people's perceptions about this disease. You have testified that you know people who fell sick and went to hospital. Those who fall sick and do not go hospital, how do they perceive breast cancer?**

Recorder: Aongee namba 4 kwanza alafu namba 2/ **let number 4 talk then number 2**

P4: Ila kutegemeana na swali mlilouliza ni kwamba kwa kweli kutegemeana na hali hiyo mi nafikiri labda ni kutokana na ukosefu wa lishe kwenye chakula kwa kweli katika kugundua tunashindwa kuelewa sana. Kwa sababu utakuta titi limeanza tu labda kunakuwa na ukosefu wa madini mwili hapo tunashindwa sana kugundua.

**But depending on the question you asked, it really depends on the situation, I think it is probably due to a lack of certain nutrients in the food, in fact we find it very difficult to understand. You find that the breast just starts swelling, maybe there is a lack of nutrients in the body, we find it difficult to know.**

M: Asante/ Thank you

Recorder: Namba 2/ **Number 2**

P2: Alafu pia mmesema mtazamo wa watu kuhusu jambo hili

**Then you have asked about perception of people on this disease**

M: Eehee/ **Yes**

P2: Mtazamo wa watu kuhusu jambo hilo kwa kweli hakuna anaeamini kuwa mtu huyo karogwa. Kwa sababu magonjwa haya ukiuliza wazee wetu wa zamani wao wanajina la ki-Ndali wanasema *imbenga.*

**In truth, people's perceptions of this subject matter, is that nobody believes that the person is bewitched. Because these diseases if you ask our Elders they identified them with names sush as Ndali vernacular langaug*e - they say imbenga.***

M: *Imbenga?.*

P2: Eeh *Imbenga*

**Yes, *Imbenga***

M: Inamaanisha nini?

**What does it mean?**

P2: Maana yake ndo kansa hiyo.

**It means cancer**

M: Kwa hiyo, kansa ya matiti inaitwa Imbenga ya matiti?

**So, is breast cancer called Imbenga of the breasts?**

P2: Zote hizo ya matiti, miguu vidonda kwa sababu kuna wengine unakuta walikuwa wanaumwa vidonda vinachimbika mpaka mfupa unaonekana. Tunaufahamu toka miaka mingi ugonjwa huu.

**All of those - ulcers in the breasts, legs; others have deep ulcers with exposed bone. We have known this disease for many years.**

M: *Imbega*

P2: Eeh

M: Akishaona kimekuwa kidonda ndo anasema hii ni Imbenga

**When it becomes a sore/ulcer it is *Imbenga?***

P2: Ndio. Kama hakitibiki sasa kama hakiponi ndo wanagundua hii ni Imbenga. Yani maana yake ndo kansa. Kansa ni kiingereza saratani Kiswahili sasa maana yake imani tuliyonayo mtazamo tunaamini kabisa huo ugonjwa hautibiki hospitali. Kwahiyo mara nyingi tunakua tunageukia miti shamba. Kwa sababu huko nyuma walikua wanafanikiwa kwenye miti shamba wanapona. Sasa waliokuwa na ujuzi wa miti shamba enzi hizo hawakurithisha huo ujuzi.

**Yes, if it is incurable (if it does not heal) then it is Imbenga. That means cancer. Cancer is an English name, in Kiswahili it is known as *Saratani. Our firm belief is that* the disease is not curable in the hospital. Therefore, we often resort to using herbs. In the past people were using herbs and they were successfully healed. Unfortunately, those who had knowledge of the medicinal plants did not bequeath that knowledge away to the next generation.**

M: Wameenda wapi sasa?

**Where have they gone nowadays?**

P2: Walishakufa sasa unakuta mtu akipata tatizo na limekuwa kubwa anaambiwa “unakwenda hospitali unasumbuka tu nenda kienyeji” unakuta sasa mtu ameshachelewa anakufa.

**They have died. When someone falls ill (sick) with cancer and the cancer is worse, the person is told “if you go to the hospital, you are just waisting your time (bothering yourself in vain); just go to the local (traditional) hebalists” Unfortunately the person delays treatment and ends up dead.**

M: Kwa hiyo, kwanza amegundua kwa kuchelewa alafu tena anamini nikienda hospitali hakuna huduma/ matibabu yake kwa hiyo anageukia kienyeji anakufa.

**So she first finds out (that she has breast cancer) when it is too late and then again he believes if I go to the hospital there is no medical treatment for her so she turns to the local herbs and eventually dies.**

P2: Ndiyo**/ Yes**

M: Sawa asante. Mwingine kuhusu hiyo.Imbenga.

**Ok, thank you. Anyone else to talk about that Imbenga?**

Recorder: Namba 7/ **Number 7**

P7: Alichosema Imbenga ni kweli tatizo lipo kwa sababu kwa mfano Imbenga inatokana na mnyama fulani kama kacheche kama paka hivi kana mkia tunasema kapenga. Kapenga anachimba shimo anaenda aidha kuchukua mahindi, njugu mawe, viazi anaenda kula mule, anapekenya kila kitu. Sasa Yule wakikuta labda Yule kapenga watu wanawinda wanakula kwa hiyo Yule kapenga akishakula wanakuta amekufa peke yake. Sisi kienyeji walivyokua wanatumia wakikuta amekufa peke yake wanachukua mfupa wa Yule kapenga ndo wanaenda kuchoma kwenye moto wakishachoma kwenye moto, wanaenda kumnyunyizia mtu kwenye kidonda kile alikua anapona ndo ilikua dawa yake anapona. Mpaka leo kama umebahatika hata kama mtu anakidonda kinataka kukuua ukishapata mfupa wa Yule kapenga ukishanyunyiza pale kinakwisha. Huo ni utaalamu wa asili.

**What he said about *Imbenga* is true. There is a problem, because for example the name Imbenga comes from a certain animal like a spark like a cat, it has a tail - we call it Kapenga. Kapenga digs a hole and goes either to pick up maize, stone nuts, potatoes and goes to eat inside the hole, eating up everything. So when hunters pass by the hole and finds the Kepenga has died by itself (without being killed), they take that kapenga's bone, take it to their home where the burn the bone and collect the ashes. Then when there happens to be a sick person with Imbenga, they treat her by sprinkling the Kapenga ashes on the sore; that was the medicine and the person recovers (heals). To these days, if you are lucky enough, if you suffer from a serious life-threatening ulcer, if you get ashes made from bone of a Kapenga and sprinkle on the wound, it heals. That is indigenous knowledge (expertise).**

M: Unausaga/unauchoma?

**Do you grind or you burn it?**

P7: Ukishachoma unausaga ule mfupa ambao Yule kapenga amekufa peke yake. Lakini ukienda kuchimba ukamkata ukachoma hauwezi ukafanya kazi mpaka ukue aliyekufa peke yake kwa hiyo ndiyo dawa ya kansa kapenga.

**After burning the bone, you grind it. It should be a bone of the *Kapenga* that has died on its own. But if you go dig and find it alive, kill it and then cut its part, and then burn it, it can't work. It has to be a bone of a Kapenga that has died on its own [not killed], that's the cure for cancer.**

M: Duuh mpaka upate amekufa peke yake sikazi kubwa.

**Duuh, It has to die on its own, isn’t that a hard task?**

P7: Ni kazi sana.

**It is a hard task indeed.**

M: Sasa swali lingine nikifuatilia swali hapo kwamba kama hivyo ndivyo, sasa unajua mpaka anagundua kwamba hii ni Imbenga anakua amechelewa na kwa hiyo haendi hospitalini. Kwa hiyo nataka kujua sababu za watu kutokugundua mapema moja ni kuchelewa anaamini kwamba huu ugonjwa hauzi kutibiwa hospitalini. Sasa nataka kujaribu kuwaulizeni aah sababu zinazofanya wasigundue mapema. Kwa sababu naona hata hao wanaogundua imeshakua kidonda wanakua wameshachelewa. Sasa ni kwa nini hawagundui mapema ni vitu gani vinasababisha kuchelewa kugundua Imbenga kabla haijafika kwenye kidonda, bado kingali hali ya ndani.

**Now another question, a follow up question, if that is the case, now you know until she discovers that this is *Imbenga* she is already late, therefore does not go to the hospital. Therefore, I want to know the reasons why people do not detect it early. One is that she believes that this disease cannot be treated in a hospital. Now I want to try to ask you aah the reasons why they do not find out early. Because I see that even those who find out after it has developed into a sore are already late. Now why don't they find out in advance? What causes the delay in discovering Imbenga before she reaches the stage of outward manisfetation in form of a wound, when the abnormality is still internal?**

Recorder Namba 1/Number

P1: Mimi nafikiri kinachochelewesha ni kwamba inapoanza huwa haitengenezi kidonda kwa hiyo baada ya kuwa sugu ndo kidonda kinaanza kinajitokeza hapo ndo mtu anagundua hii inawezekana ni kansa.

**I think what causes the delay is that when the ulcer begins to show up, it does not form a serious sore, it is until when it becomes chronic and that is when one realizes this is probably cancer.**

M: Anhaa. Mwingine karibu.

**Anhaa, Another partipant? Welcome!**

Recorder: Namba 2/ **Number 2**

P2: Pili ni kwamba kinachochelewesha mtu asigundue mapema anapopatwa na kidonda kile sawa anaaenda hospitali. Hospiali wanamtibu lakini kile kidonda bado kinaendelea. Anarudi hospitali anaenda hospitali karibu mwaka mzima na hakiponi ndo anagundua hiki ni kansa kwa sababu kwa nini hakiponi kwa madawa ya hospitali?

**Secondly, what delays a person from detecting it early is that when she gets a sore she goes to the hospital. Hospitals treat her but the wound is still developing. She goes back to the hospital for almost a year and does not heal (recover) and she later discovers that this is cancer because the wound has not healed from the hospital medication.**

M: Anhaa

P2: Eeh sio kwamba amechelewa amekaa na kidonda kile nyumbani kidonda kinamsumbua anaenda hospitali anapewa dawa za vidonda. Anarudi anakaa siku ngapi kinarudi tena kidonda kile mwisho wake ndo anaambiwa kidonda hiki kwa kuwa hakiponi tatizo hili ni kansa.

**Eeh not that she is late because she is sitting at home. The ulcer (sore) is bothering her and she is going to the hospital to get medication. Unfortunately, she goes back to the hospital after few days after the ulcer re-occurs. At the end, becsue the ulver is recurring; she is told the sore is cancer.**

M: Kwa hiyo hata kwenye matiti ni hivyo hivyo?

**So the same applies to the breasts.**

P2: Eeh/ **Yes**

M: Anaenda hospitali anapewa dawa anarudi karibu mwaka mzima.

**She goes to the hospital and is given medicine, no improvent, so she returns to the hospital for almost the whole year.**

P2: Yah/ **Yes**

M: Mwenye mawazo tofauti ya kuongeza hapo?

**Anyone with different ideas to add here?**

P: Lakini pia tuseme hii kansa inawezekana kusema kuna mgao inategemeana na damu. Kwa sababu unakuta mwingine kidogo tu hata kakitu kamekukwaruza imekua kansa. Yani unakuta mwingine labda kamti kamekukwaruza unaenda kutibiwa unakuta kanaendelea kanaendelea tu ndo unajua hii ni kansa. Kwa hiyo inawezekana tukasema inategemeana pia na damu ya mtu jinsi ilivyo. Kwa sababu hata mwanaume mwingine na sisi tuna kansa tunashikwa na kansa ndo maana unakuta tunashambuliwa na vidonda mara nyingi. Lakini kwenye matiti sio sana unakuta kwenye vidonda au sehemu Fulani ya siri au nini kwa hiyo ile nyingine unaweza ukakuta akishakwaruzwa na kitu Fulani kakamchoma unakuta hata kama ni mwiba unakuta tayari ule mguu unaendelea kuwa kansa. Kwa hiyo ni nadhani inategemeana na hali Fulani damu ya mtu.

**Also, we may say that cancer is likely to be blood-dependent. Because you find someone who gets something like a minor scratch to his or her body, it grows into a cancer. I mean you find another person maybe gettin a bruising from a small yardstick that grows into cancer. Then you end up going to the hospital for treatment repeatedly and eventually you realize this is cancer. Therefore, it is possible to say that it also depends on one's blood type. Because even men are prone to cancer because they too may find themselves dealing with ulcers (sores). However, in the men’s breast it is not much. You find sores in the genitals or in the legs after getting a scratch. Therefore, I think it depends on the particular condition of a person's blood.**

M: Asante. Sasa kuna hii huduma tulikua tunauliza wakati ule tulipokuwa tunaongea na mtu mmoja mmoja. Kitu kinaitwa uchunguzi binafsi wa matiti aah. Kwamba mama mwenyewe anajichunguza anajishika mwenyewe amajipapasa vizuri kabisa na kuweza kugundua kwamba kuna tatizo au hakuna tatizo akigusa au akihisi kama kuna kidude ndani ambacho kama kijivimbe au nini ambacho kinaashiria kunaweza kuwa na mwanzo wa Imbenga kuweza kutokea. Sasa huo uchunguzi binafsi ni mkakati au ni namna ya kufanya ambayo kama mama anafanya itamwezesha kugundua mapema kabla hakijawa kidonda. Kama anatabia ya kufanya hivyo inamsaidia kabla hakijawa kidonda sasa hapa kwenu, wamama wanafanya hivyo? Yani kina mama wanahiyo tabia ya kuwa na muda wanajichunguza wenyewe anajiangalia titi jinsi lilivyo ananza kulishika, kulipapasa vizuri eh na kuona kama kuna chochote kama kijiuvimbe chenye kuashiria saratani. Huwa wanatabia ya kufanya hivyo?. Sassa tuanze na wake zenu nyie wenyewe alafu sasa muende kwa upana zaidi, Karibu.

**Thank you. Now, we were talking about a service [breast exam screening], when we were interviewing you individually, something called self-examination of the breasts aah. That the mother herself examines her breasts by touching her breasts very well and being able to detect if there is a problem or no problem - by touching or feeling if there is something inside the breasts like a lump or that something may indicate a beginning of *Imbenga*. Now that self-examination is a strategy or a way of doing it. If the mother does so self-examiniation, she can be able to detect if there is a problem early before it becomes an ulcer (open sore). Now here at** [masked for confidentiality]**, do mothers do it (self-exams)? Now let's start with your own wives and then go broader. Welcome**

Recorder: Namba 7/ **Number 7.**

P7: Mi nadhani mi naweza nikasema hawana taratibu za namna hiyo wala mazoea yoyote. Lakini nadhani kwa elimu hii mnayotoa tunaweza tukapata maelekezo kuwaelimisha. Mimi nilifikiri kwamba inakua siku moja mnawaita kina mama na kina baba mnakaa pamoja mnatoa elimu sisi na wake zetu linakua jambo la msingi sana.

**I think, I can say they do not engage in such procedures nor such habits procedures or any procedures. But I think with this education you are providing we can get instructions how to educate them. I thought that one day you would call mothers and fathers, we sit together and educate our wives and us, it would be a very fundamental thing.**

M: Kwa hiyo kwa sasa hivi hawafanyi.

**So for now they do not do breast self-examination**

P7: Hamna, hamna kwa kweli. Kwa elimu inawezekana mkitusaidia tunaweza tukawaelimisha wakaelewa wakafanya uchunguzi.

**No, not really. With education, it is possible if you help us we can educate them, they will understand and do self-examination (of the breast).**

M: Sawa asante. Wengine mnasemaje, mnakubaliana kwamba hawafanyi kwa sasa uchunguzi binafsi was matiti?.

**Okay thank you. What do others say? Do you agree that they are not currently conducting self-examination?**

Pwote/All: **Hawaafanyi hawafanyi./ They do not do**

M: Je vipi kuhusu aah sababu za kutofanya mumesema kwanza ni elimu.

**What about the reasons for not doing aah, you have said number one is education.**

Pwote: Yes

M: Kingine?, sababu za kutofanya?

**What else?, Reasons for not doing?**

P: Ni elimu tu hakuna kingine?.

**It's just education and nothing else?**

M: Anhaa hakuna labda imani fulani au mila na desturi - kwamba labda kujishikashika titi sio sawa. Hakuna imani kama hizo?.

**Is there probably no particular belief or tradition - that maybe people believe that self-palpation of the breast is not the right thing to do. Are there no such beliefs?**

Pwote: Hamna

**No**

M: Je hospitalini? huduma hii ya kuchunguza matiti, lakini inayofanyika hospitalini, wamama wa hapa wamekua wakienda hospitalini kwa ajili ya kufanyiwa huduma hiyo ya kuchunguzwa matiti? Sio kwamba ana kidonda, hapana. Anaenda tu daktari akathibitishe kama yupo sawa au kuna tatizo. Anaenda tu kabla hajaanza kusikia dalili yoyote, hamna kidonda hamna uvimbe lakini anaenda tu hakuna uvimbe wa wazi wa nje yeye anaenda tu daktari akachunguze kama kuna chochote kisicho sawa basi daktari atachukua hatua za kitabibu.

**This breast examination {clinical breast examination), which is done at the hospital, do women access it? Not because they have a sore though. She just goes to the doctor to confirm if she is OK or there is a problem [screening]. She just goes [to be screened] before she starts feeling any symptoms, there is no sore no swelling but she just goes. There is no obvious external swelling, she just goes to the doctor to check if there is anything abnormal then the doctor will take appropriate medical action.**

Recorder: Namba 1/ **Number 1**

P1: Hapana/ **No**

M: Hawaendagi

**They do not go**

P1: Hawaendi eeh labda mpaka mtu atakapojisikia maumivu ila hivi hivi hapana.

**They do not go eeh maybe until someone feels pain, but just apparently they do not go.**

M: Anhaa hawaendi mpaka mtu atakapojisikia maumivu

**They do not go until someone feels pain.**

P1: Eeh./ **Yes**

M: Ila wasiposikia maumivu hawaendi

**But when they do not feel pain they do not go.**

P1: Eeh/ Ndiyo/ **Yes**

M: Mnakubaliana ndivyo ilivyo?

**You are all agreeing that that is how it is.**

Pwote: Ndivyo ilivyo

**Yes. That is how it is.**

M: Anhaa. Sawa na sababu za kutoenda kwa ajili ya upimaji wa matiti mojawapo mmesema ni elimu. Kingine? kwa nini hawaendi kwa ajili ya uchunguzi wa mwanzo daktari akawachunguze? Karibu.

**Anhaa. Okay, the reasons for not going for breast examination - one of them, you have said, is education [lack of]. Other reasons? why don't they go for a preliminary examination and the doctor examines them? Welcome.**

Recorder: Namba2/ **Number 2**

P2: Na tabia yetu tu sisi wa afrika usipoumwa hatuendi hospitali. Kwamba nimeondoka tu asubuhi nipo fiti tu naenda hospitali nasema wakanifanyie uchunguzi aah sijawahi kuona mtu yoyote akifanya hivyo aah sijawahi kuona.

**And our behaviour, we Africans when we are not sick, we don't go to the hospital. That I just leave home, I am physically fit and I decide to leave in the morning and go to the hospital and say “let them examine me” aah I have never seen anyone do that aah I have never seen.**

M: Tabia yetu Waafrika?

**African behaviour?**

P2: Eeh/ **Yes**

M: Hatufanyi/ **we do not do.**

Recorder: Namba 5/ Number 5

P5: Ninavyoona hatuwezi kwenda hospitali na uchumi wetu unachangia. Sisi tukiona unajisikia tu vizuri, unakula upo salama hauwezi ukaenda unasema tu Mungu nashukru. Kwa sababu uchumi wetu huku vijijini uko chini. Kuamua kwamba nikachunguzwe itakuwa ni kama ninajichelewesha nisiende kwenye kazi zingine.

**As I see it, we cannot go to the hospital and our economy is contributing to this fate. When we feel okay, we can eat, we are safe, we cannot just to the go (to the hospital), you just say God thank you. Because our rural economy is in shambles, deciding whether to go to a healthfacility for check-up is not easy, it will delay my other job (work) obligations.**

M: Anhaa, kwa hiyo anaona kwamba kwenda kwenye hospitali wakati hajisikii dalili yoyote ni sawa na kupoteza muda wa kwenda kufanya kazi. Lakini pia pengine ataenda kutumia pesa wakati anaona nikalipe pesa kwa kitu ambacho sijasikia maumivu wala changamoto yoyote.

Pwote/All: Ndio/ **Yes**

M: Anhaa sasa tumezungumzia kwamba wanawake hawafanyi. Je wanaume wa hapa ambao mko kwenye ndoa je wanafanya hiyo huduma kusaidia wake zao kufanya huduma hiyo kugundua mapema, wanaume. Naona vichwa vinatikiswa, maana yake hawafanyi.

**Anhaa now we have talked that women do not do [breast self-examination]. Are the men here who are married doing the service of helping their wives to do breast self-examination so they can detect breast cancer early, men? I see the heads are shaking, which means they don’t.**

P: Ni kweli tuseme ukweli hatufanyi. Hii ndo imekuwa mara ya kwanza kutuelimisha eeh mimi ila nilishawahi kusikia mama akishikwa matiti mara kwa mara kuna kitu huwa kinajitokeza kwenye matiti. Hilo niliwahi sikia lakini sina uhakika kwamba iliwahi tokea kwamba ukimshika shika sana mama matiti kwamba inatokea tatizo fulani kwenye matiti. Sasa sikuelewa kwamba ni tatizo gani labda kwa kuwa mmekuja mtatusaidia kama kuna kitu cha aina hiyo, nina imani mnaweza mkawa na majibu ya kutusaidia. Lakini hatuna mazoezi yoyote. Mtu akisema kweli “mi huwa nasaidia” atakuwa ni muongo hatujafanya hivyo hatufanyi hivyo.

**It is true to say the truth we do not. This is the first time we have been educated. However, I have heard that when the woman’s breasts are palpated several times something happens in the breasts. I have heard that but I am not sure if it ever happened that if you palpate the woman’s breasts frequently, something happens in the breast. Now I do not understand what the problem is maybe because you have come you will help us if there is such a thing and I trust you can have answers to help us. But we don't have any practice [of helping the spouse do breast self-examination] because if someone says “I always help” he will be telling lies, we have not done that, we do not do that.**
